# The consequences of debris flows in Brazil: a historical analysis based on recorded events in the last 100 years

**DOI:** 10.1007/s10346-022-01984-7

**Published:** 2022-12-13

**Authors:** Victor Cabral, Fábio Reis, Vinicius Veloso, Claudia Correa, Caiubi Kuhn, Christiane Zarfl

**Affiliations:** 1 Applied Geology Department, Institute of Geosciences and Exact Sciences - IGCE, São Paulo State University – UNESP, Av. 24A, São Paulo, Rio Claro 1555 Brazil; 2 Geo- Und Umweltforschungszentrum (GUZ), University of Tübingen, Schnarrenbergstraße 94 – 96, Tübingen, Germany; 3 Center for Geosciences Applied to Petroleum (UNESPetro), São Paulo State University – UNESP, Av. 24A, São Paulo, Rio Claro 1555 Brazil

**Keywords:** Landslide database, F-N curve, Mortality rate, Debris flows, Natural disasters

## Abstract

This study aims at providing an overview of the socioeconomic consequences that debris-flow events have caused in Brazil, positioning the country in the international scenario and identifying areas where targeted actions are necessary. The analysis is conducted by calculating the debris-flow mortality rate (MR) and by using the so-called F-N plots (frequency of events that have caused *N* or more fatalities vs. the number of fatalities), based on a compilation of debris-flow-related disasters from 1920 to 2021. In total, 45 debris-flow events were documented in the considered period, responsible for 5771 fatalities and more than 5.5 billion USD in economic losses. The Serra do Mar Mountain Range is the main site of reported debris-flow occurrences (64.5%), followed by Serra da Mantiqueira (13.3%), and Serra Geral (13.3%). Southeast Brazil (SEB) is the region most affected by debris-flow events, due to the highest population density and the development of several cities in hilly areas, such as Petrópolis (Rio de Janeiro state) and Cubatão (São Paulo state). The debris-flow MR of SEB is higher than any other region in Brazil, pushing the national debris-flow MR upwards, and the F-N curve of SEB consolidates the region as the one with the highest risk to the phenomenon, indicating a higher probability of fatal events. The F-N plots further show that debris-flow events in Brazil represent a higher societal risk than in countries such as China, Japan and Italy. While there are differences in country size and the scale effect should be considered, these results highlight the urgent need for investments in disaster prevention and preparedness programs.

**Supplementary Information:**

The online version contains supplementary material available at 10.1007/s10346-022-01984-7.

## Introduction

A natural hazard occurs when processes of the geophysical environment have the potential to cause damage or loss to a vulnerable human community (Stillwell 1992; Alcántara-Ayala 2019); when their consequences have major negative impacts on society, they become natural disasters (Burton and Kates 1963; Alcántara-Ayala 2002). Economic, political, and social factors of countries of the Global South^1^ can contribute to increasing their vulnerability to natural hazards, which results in higher number of fatalities and loss of infrastructures when compared to developed countries (Devoli et al. 2007; Patel and Burke 2009).

Exogenous processes (e.g., floods, landslides, snow avalanches) are some of the most commonly occurring phenomena that negatively affect humans and infrastructures worldwide (Kahn 2005; Alcántara-Ayala 2019). In Brazil, hydrogeomorphic processes, triggered by high-intensity precipitation, are the most recurrent and deadly among the natural disasters (Kahn 2005; CEPED/UFSC 2013; Assis Dias et al. 2018; Kobiyama et al. 2019). The increasing frequency of extreme and high-intensity rainfall events due to global warming has been associated with an increase in the frequency and magnitude of floods and landslides in Brazil (Cavalcanti et al. 2017; Marengo et al. 2021), as well as worldwide (Fowler and Hennessy 1995; O’Gorman and Schneider 2009; Westra et al. 2014; Deng et al. 2021), which highlights the importance of risk and vulnerability studies.

Approximately 9 out of 100 people in Brazil live in disaster-prone areas, with landslides and debris flows associated to a higher number of fatalities per event (Kahn 2005; Alcántara-Ayala 2019). Between 2000 and 2010, landslide and debris-flow events caused approximately 1700 deaths in the country, affected almost 8 million people and caused economic losses of 1.5 billion USD (Bastos et al. 2015). In 2011, high-magnitude landslides and debris flows in the state of Rio de Janeiro officially caused more than 942 deaths and 1.4 billion USD in losses, being considered the 8th worst landslide event in world history (Assis Dias et al. 2018; Rosi et al. 2019). The 2011 catastrophe led to the creation of the National Center of Monitoring and Early Warning of Natural Disasters (*Centro Nacional de Monitoramento e Alertas de Desastres Naturais* – CEMADEN), an important advance towards natural disasters prevention and monitoring in Brazil.

With the creation of CEMADEN in 2011, the national disasterss database (*Sistema Integrado de Informações sobre Desastres –* S2ID), managed by the Ministry of Regional Development (*Ministério do Desenvolvimento Regional* – MDR), was also established to document natural disasters. Prior to S2ID, no centralized record of natural disasters at a federal level occurred, with states having autonomy over disasters cataloguing. A national disasters database is crucial in risk assessment studies and in the understanding of the underlying dynamics of a phenomenon, helping to reduce and mitigate associated damage (Bollschweiler and Stoffel 2010; Wirtz et al. 2014).

The comprehension of the impact that a specific natural hazard represents in a country is a basic step in their management and monitoring, as highlighted by the targets of the Sendai Framework for Disaster Risk Reduction (2015–2030, UNDRR 2015). The continental dimension of Brazil and the heterogenous management of natural disasters are a great challenge for the implementation of a thorough database, as different states implement different budgets for disaster monitoring and prevention (CEPED/UFSC 2013). An effective database should cover a long period of time with reliable information about the socioeconomic losses (Borden and Cutter 2008), and a consistent documentation of natural disasters in Brazil is recent (from 1991) when compared to European and North American countries (e.g., Italy: Guzzetti 2000; Germany: Damm and Klose 2015; USA: Mirus et al. 2020).

Among the landslide types, debris flows are associated to a specifically high damage per event due to their high impact force, velocity, and high sediment content per unit volume (Coussot and Meunier 1996; Takahashi 2006). Debris flows are characterized by bulk densities that vary from 1800 to 2300 kg m^−3^, velocities that range from 3 to 25 m s^−1^, and sediments contents of 40 to 80%, which can range in size from organic matter to large (> 5 m) rock boulders (Costa 1988; Takahashi 2006; Iverson et al. 2011; Zhuang et al. 2015; Santangelo et al. 2020). In Brazil, debris flows are mainly initiated by rainfall-triggered landslides (Wolle and Hachich 1989; Lacerda 2007) and their rheological characteristics vary according to the geology and geomorphology of the catchments (Lacerda 2007). Moreover, debris floods, flash floods, and floods are some of the phenomena that often occur in association to debris flows, especially during large magnitude events (Wilford et al. 2004; Dowling and Santi 2014; Hungr et al. 2014; Church and Jakob 2020).

Increasing urbanization in mountain regions, especially in south and southeast Brazil, has been highlighted by recent studies (Guerra et al. 2007; Patel and Burke 2009; Londe et al. 2018), which increases the exposure to potential debris-flow related damage, as changes in society and economic development are the main driving forces that can magnify associated losses to a natural phenomenon (Andres and Badoux 2018). In this context, the aim of this study is to quantitatively estimate the socioeconomic consequences that debris flows have caused in Brazil, by calculating the phenomenon’s mortality rate and by applying F-N plots (frequency of events that have caused *N* or more fatalities vs. the number of fatalities), based on the compilation of historical events that have caused fatalities and/or economic losses between 1920 and 2021. The debris-flow event cataloguing is conducted based on different disaster databases, both worldwide and national, on scientific publications and on governmental and journalistic reports. This catalogue is seen as a contribution to the collaborative effort among the scientific community to provide basic debris-flow data for a long-term hazard and risk analysis.

## Methods

### Debris-flow data collection

Two main data-source types are used in the compilation: disaster databases, both national and international, and technical and scientific documents that describe debris flows in Brazil, including journalistic reports. The data gathered from these sources are combined to create a new catalogue, which considers the following attributes: date of the event, trigger time, mountain range, catchment, city, state, triggering-event intensity (i.e., rainfall in mm), magnitude (i.e., volume of sediments mobilized by the debris flow, in m^3^), human losses (fatalities, injuries, missing, homeless, displaced), material losses (houses, public and private buildings, public infrastructures) and economic losses (in United States Dollar—USD). Economic losses are estimated by using the conversion rate of the date of the event from Brazilian currency to USD, according to the reports of exchange rates of the US Treasury. The values were then adjusted to inflation (www.usinflationcalculator.com).

Varnes’ (1978) landslide classification, updated by Hungr et al. (2014), is adopted in our analyses to identify debris-flow events. The Brazilian Code of Disasters (COBRADE) also adopts Varnes’ (1978) classification, adapted by Augusto Filho (1992). Although, officially, Brazilian databases follow international standards (CEPED/UFSC 2013), COBRADE differs from the international peril terminology and classification proposed by the Center for Research on the Epidemiology and Disasters (CRED), used in international databases. Landslides are classified as hydrological disasters by CRED (when triggered by rainfall and snow melt) and as a geological disaster by COBRADE. Moreover, CRED classifies debris flows as landslide events, which is not the case for COBRADE (see online resources for the comparison of definitions).

#### Databases

The databases used in this work are the Brazilian disaster database (S2ID, Brasil 2021a), the International Disasters Database (EM-DAT 2021) and the databases maintained by some research institutes of the federate states. It is important to note that S2ID exclusively documents a disaster when a city or state declares state of emergency (CEPED/UFSC 2013). Similarly, in the EM-DAT, a natural disaster is only documented when a state of emergency is declared and more than 10 fatalities are reported or 100 people are affected (Van den Eeckhaut and Hervás 2012; CEPED/UFSC 2013).

The S2ID is the official database for natural disasters in Brazil since January 2013 (https://s2id.mi.gov.br). It systematically documents disasters that occurred in the country from 1991 onwards, although more expressive disasters prior to 1991 are also documented inconsistently. The identification of a disaster in the platform is conducted since 2013 via the “Identification of Disasters Form” (FIDE) and, prior to 2013, through either the “Preliminary Notification of Disasters Form” (NOPRED), the “Damage Evaluation Form” (AVADAN) or journalistic reports. These forms and reports are available for events from 1940 to 2016 in the S2ID platform, with those after 2016 having to be requested privately via the Fala.Br platform, which is the official channel to request public information from government institutions. The distinction between landform processes was made based on photographs and the description provided in the database, as well as with the aid of publications and news reports.

The International Disasters Database (EM-DAT 2021) is developed and operated by CRED, within the Université Catholique de Louvain (Belgium) (https://public.emdat.be). The database is based on information retrieved from United Nations (UN) agencies, official governmental offices, the International Federation of Red Cross and Red Crescent Societies (IRFC), research organizations, insurance periodicals and reinsurance publications (Wirtz et al. 2014). EM-DAT provides a more complete documentation of Brazilian disasters when compared to other freely available international databases.

The state of São Paulo also catalogues disasters in the state via the Geological Institute (IG). IG systematically documents disasters since 1991, even those for which no casualties are reported, based on journalistic articles and the on-site activities of the authorities during disasters response, granting a more thorough depiction of the landslide and debris-flow consequences in the state. Similar database for the other 26 states was not available according to their Civil Defense departments, with their disasters data accessible only through S2ID.

#### Publications

Since no official documentation of landslides and debris-flow events prior to 1991 is available in a consistent and systematic manner, technical reports and scientific documents that describe and compile debris-flow events in Brazil since 1920 were also analyzed. Data from debris-flow publications were mainly retrieved using bibliographic search tools: SCOPUS, from Elsevier (2022); Web of Science, from Clarivate Analytics (2022); and “Periódicos CAPES,” from the Brazilian Research Agency “*Coordenação de Aperfeiçoamento de Pessoal de Nível Superior*” (CAPES). The keywords used, both in English and Portuguese, were “landslides” (*escorregamentos*), “debris flows” (*fluxos de detritos*), “mudslides” (*fluxo de lama*), “flash floods” (*enxurrada*) and “floods” (*inundação*).

A total of 7638 publications were accessed and analyzed for data compilation on debris flows. Among those, 4% of the publications were identified by the keywords “debris flows” and “*fluxo de detritos*” (see online resources for the table with the bibliographic search results). Studies about floods, flash floods and landslides were analyzed due to the close relationship between these processes and debris flows in mountain areas of Brazil, as well as in other parts of the world (Hungr et al. 2014).

A good review of Brazilian debris-flow events was made by Kobiyama and Michel (2015) and is also used as a reference. The Kobiyama and Michel (2015) study based the creation of an incipient debris-flow database by Kobiyama et al. (2015) and Kobiyama et al. (2019), which included the year and location (municipality) of the event, as well as the number of fatalities. Dowling and Santi (2014) also conducted a comprehensive compilation of debris-flow events worldwide, encompassing events from 1950 to 2011 and describing the magnitude, triggering-event intensity, as well as the number of fatalities and economic losses. In Dowling and Santi’s (2014) database, however, only high-magnitude Brazilian debris-flow events are documented.

Furthermore, for each event identified in the bibliographic search and in the disaster databases, different sources of information for the same debris-flow event were compared to more accurately depict the extent of the associated damages. A great challenge during cataloguing was mismatched information between sources, which challenged an accurate estimation of the real damage. When uncertainties occurred, information from peer-reviewed articles was favored, followed by governmental reports, S2ID, EM-DAT and journalistic reports. When mismatched information from the same type of source was found, the worst-case scenario was adopted, i.e., the highest number of fatalities or largest economic losses. Data scarcity in disasters documentation, especially of economic losses, is observed across all different sources of information, even for more recent events.

### Analysis of the societal impact

Based on the compiled catalogue of debris-flow events, the consequences that the phenomenon have caused in Brazil was estimated based on the calculation of the mortality rate (MR), as well as by the relationship between the frequency of events and their fatality rate, using the so-called F-N plots.

Calculating the MR is a direct method of estimating the debris-flow impact on a country and is expressed as the number of deaths by debris flows per a specified population size in a defined period of time, e.g., a specific year (Guzzetti 2000). Here, the MR is calculated per 100,000 people (Evans 1997).

Another method to estimate the impact of debris flows to the society is the application of F-N plots. F-N plots provide the likelihood of multiple fatalities due to a debris-flow event, by plotting the cumulative frequency of events that have cause *N* or more fatalities (*F*) with the number of fatalities (*N*), in a log–log scale (Fell and Hartford 1997). Following Ball (1998), the equation for the F-N criterion can be represented as:

$$F=k*{N}^{-a} (1)$$

With *F* as the cumulative frequency of events with *N* or more fatalities, *N* is the number of fatalities, *a* is the aversion factor, and *k* is a constant. The slope of F-N curves is an indicative of the risk of a country or location is under, with steeper slopes indicating a lower frequency of high-magnitude events when compared to curves with gentler slopes (Ball 1998).

## Results

**Fig. 1:**
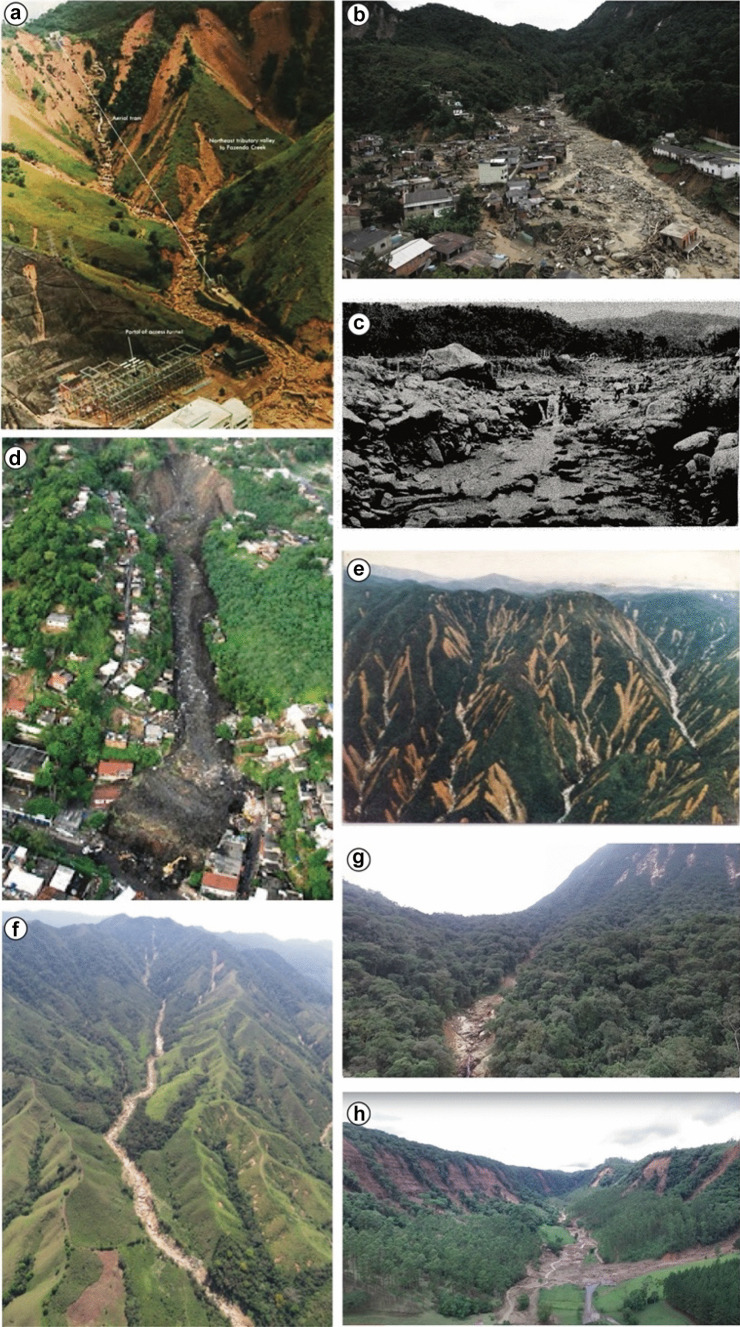
Debris-flow events in Brazil. **a** Serra das Araras (Piraí, Rio de Janeiro state) in 1967. Mudslide in the Fazenda catchment, one of many that destroyed the city. Picture from Jones (1973). **b** Teresópolis 2011. Generalized landslides triggered debris flows in several catchments. Picture from Oliveira Filho (2012). **c** Caraguatatuba 1967. In the picture, large rock boulders are deposited at the Santo Antonio catchment outlet. Picture from Cruz (1975). **d** Niterói 2010. Debris avalanche at a deactivated landfill area, which caused the death of 48 people and 221 missing. Picture from Estadão (2010). **e** Cubatão 1985. Generalized landslides at the Serra do Mar hillslopes that triggered high-magnitude debris flows at the region’s catchments. Picture from IPT (1988). **f** Itaoca 2014. Landslides in the headwaters’ region triggered a high-magnitude debris flow. Picture from Gramani and Arduin (2015). **g** Guaratuba 2017. Landslides in the headwaters’ region triggered a high-magnitude debris flow (120,000 m^3^). Picture from Cabral et al. (2021). **h** Vale do Itajaí (Ibirama city) 2020. Generalized landslides triggered high-magnitude debris flows, debris floods, and flash floods. Picture from youtube video: “Um vale de destruição!” uploaded by Vale Agrícola, 19 Dec. 2020, https://www.youtube.com/watch?v=xH900IPzgow

According to our catalogue, 45 debris-flow events occurred in Brazil between 1920 and 2021, which have caused fatalities and/or economic losses. Table 1 shows the characterization of each event and Fig. 1 shows pictures of some of the country’s most expressive debris flows. The full editable and updatable version of the database is available as a Supplementary Information in online resources, with the inclusion of the references that contain a more complete description of the events for the purpose of this study.

**Table 1:** Debris-flow database, catalogued based on different sources. *Inj*, injured; *Mis*, missing people; *Hom*, homeless; *Dis*, displaced; *Infra*, infrastructures; *N/A*, not available

**Date**	**Time (GMT -3)**	**Mountain range**	**Catchment**	**Location**	**State**	**Rainfall intensity**	**Magnitude** **(m**^**3**^**)**	**Human losses**					**Material losses**			**Economic losses (USD)**
								**Death**	**Inj**	**Mis**	**Hom**	**Dis**	**House**	**Building**	**Infra**	
10.03.1928	05:00	Serra do Mar	Monte Serrat Hills	Santos	São Paulo (SP)	N/A	130,000	81	7	N/A	N/A	N/A	15	01	N/A	24,112,000
15.12.1948	N/A	Serra da Mantiqueira	Angu, Aventureiro, Pirapetinga, Pomba	Region of Volta Grande	Minas Gerais (MG)	400 mm / 24 h	> 10^6^	250	N/A	N/A	N/A	N/A	60	N/A	3	N/A
01.03.1956	18:00	Serra do Mar	Monte Serrat Hills	Santos	São Paulo (SP)	954 mm / month	N/A	21	102	N/A	N/A	N/A	50	N/A	1	N/A
11.01.1966	N/A	Serra do Mar	Guanabara bay Hydrographic basin	Rio de Janeiro	Rio de Janeiro (RJ)	245 mm / 24 h	N/A	250	1000	N/A	18910	39203	1000	N/A	> 100	75,547,344
11.01.1966	N/A	Serra do Mar	Quitandinha	Petrópolis	Rio de Janeiro (RJ)	327 mm / 72 h	N/A	45	35	N/A	N/A	N/A	N/A	N/A	N/A	
26.03.1966	N/A	Serra do Mar	N/A	Petrópolis	Rio de Janeiro (RJ)	188 mm / 24 h	N/A	40	N/A	N/A	N/A	N/A	N/A	N/A	N/A	N/A
20.01.1967	N/A	Serra do Mar	Guanabara bay Hydrographic basin	Rio de Janeiro	Rio de Janeiro (RJ)	181 mm / 24 h	N/A	200	300	N/A	N/A	N/A	1	2	N/A	N/A
22.01.1967	23:00	Serra do Mar	Ribeirão da Floresta, Ribeirão das Lajes	Serra das Araras (Piraí)	Rio de Janeiro (RJ)	114 mm / hour	> 10^6^	300	N/A	1400	N/A	> 10000	> 100	1	1	N/A
18.03.1967	15:00	Serra do Mar	Santo Antônio, Guaxinduba, Pau d'alho, Canivetal, Camburu	Caraguatatuba	São Paulo (SP)	420 mm / 24 h	7,600,000	436	N/A	N/A	3000	N/A	400	N/A	N/A	63,449,964
27.04.1971	N/A	Salvador Fault Hills	Miolo	Salvador	Bahia (BA)	368 mm / 24 h	N/A	104	2000	N/A	N/A	7000	1400	N/A	N/A	34,884,425
27.02.1971	N/A	Serra do Mar	Ultrafértil	Cubatão	São Paulo (SP)	N/A	N/A	N/A	N/A	N/A	N/A	N/A	0	1	N/A	N/A
18.08.1972	08:15	Serra da Mantiqueira	Piracuama	Campos do Jordão	São Paulo (SP)	N/A	70,000	17	NA	N/A	N/A	1000	80	1	N/A	N/A
23.03.1974	N/A	Serra Geral	Tubarão river basin	Tubarão	Santa Catarina (SC)	205 mm / 24 h	N/A	40	N/A	N/A	N/A	32500	N/A	N/A	N/A	666,840,081
29.04.1974	N/A	Serra de Maranguape	Pirapora	Maranguape	Ceará (CE)	N/A	N/A	91	N/A	NA	N/A	N/A	N/A	N/A	N/A	N/A
25.12.1975	N/A	Serra do Mar	Grota funda	Cubatão	São Paulo (SP)	248 mm / 24 h	> 10^5^	0	N/A	N/A	N/A	N/A	N/A	N/A	1	N/A
28.01.1976	N/A	Serra do Mar	Braço norte	Cubatão	São Paulo (SP)	40 mm / hr	100,000	0	N/A	N/A	N/A	N/A	N/A	1	N/A	N/A
23.01.1985	N/A	Serra do Mar	Perequê, Mogi	Cubatão	São Paulo (SP)	84 mm / h	> 10^6^	0	N/A	N/A	N/A	N/A	N/A	1	1	N/A
12.1986	N/A	Serra da Mantiqueira	Braço	Lavrinhas	São Paulo (SP)	70 mm / h	160,000	11	N/A	N/A	N/A	N/A	N/A	N/A	N/A	2,329,412
22.01.1988	N/A	Serra do Mar	Rio das Pedras	Cubatão	São Paulo (SP)	25 mm / h	N/A	10	N/A	N/A	N/A	N/A	10	N/A	N/A	N/A
05.02.1988	N/A	Serra do Mar	Cuiabá, Quitandinha	Petrópolis	Rio de Janeiro (RJ)	384 mm / 48 h	N/A	171	600	N/A	N/A	5000	1100	N/A	N/A	N/A
19.02.1988	N/A	Serra do Mar	N/A	Rio de Janeiro	Rio de Janeiro (RJ)	230 mm / 24 hs	N/A	289	734	N/A	N/A	18560	N/A	N/A	N/A	935,000,000
19.05.1989	N/A	Salvador Fault Hills	Central area of the city (Miolo)	Salvador	Bahia (BA)	83 mm / 24 h	N/A	69	30	N/A	N/A	N/A	N/A	1	N/A	N/A
14.10.1990	03:00	Serra do Mar	Itajaí river basin	Blumenau region	Santa Catarina (SC)	89 mm / h	N/A	17	764	N/A	N/A	754	66	N/A	N/A	N/A
18.03.1992	13:40	-	Vila Barraginho (old landfill)	Contagem	Minas Gerais (MG)	19.3 mm / 24 h	30,000	36	70	N/A	N/A	3000	300	N/A	N/A	N/A
13.02.1996	N/A	Serra do Mar	Quitite, Papagio, Pau da Fome, Vale Encantado, Travessa do alemão, Rio das Pedras	Rio de Janeiro	Rio de Janeiro (RJ)	202.5 mm / 24 h	> 400,000	65	65	N/A	N/A	N/A	500	1	N/A	N/A
06.02.1994	N/A	Serra do Mar	RPBC	Cubatão	São Paulo (SP)	60 mm / h	300,000	0	N/A	N/A	N/A	N/A	N/A	1	N/A	73,332,253
23.12.1995	16:30	Serra Geral	Rio Figueira	Timbé do Sul	Santa Catarina (SC)	176 mm / 24 h	N/A	10	N/A	6	250	580	N/A	N/A	N/A	71,311,286
14.02.1996	N/A	Serra do Mar	N/A	Ubatuba	São Paulo (SP)	359.4 mm / 24 h	N/A	11	N/A	N/A	300	N/A	30	N/A	N/A	N/A
09.04.1996	N/A	Serra do Mar	RPBC	Cubatão	São Paulo (SP)	18 mm / h	16,000	0	N/A	N/A	N/A	500	0	1	N/A	0
12.12.1999	N/A	Serra do Mar	km 42, Anchieta Highway	Cubatão	São Paulo (SP)	128 mm / 24 h	300,000	0	N/A	N/A	N/A	N/A	N/A	N/A	1	N/A
01.01.2000	N/A	Serra da Mantiqueira	Seco stream	Lavrinhas	São Paulo (SP)	55 mm h-1	N/A	N/A	N/A	N/A	N/A	N/A	N/A	N/A	N/A	N/A
01.01.2000	N/A	Serra da Mantiqueira	Morro do Britador	Campos do Jordão	São Paulo (SP)	400 mm / 96 h	N/A	10	N/A	N/A	N/A	N/A	300	N/A	N/A	N/A
24.12.2001	16:18	Serra do Mar	Cuiabá, Araras	Petrópolis	Rio de Janeiro (RJ)	300 mm / 24 h	N/A	45	29	22	777	4000	N/A	113	N/A	190,757,690
23.11.2008	10:00	Serra do Mar	Itajaí river Basin	Ilhota, Brusque, Blumenau region	Santa Catarina (SC)	337 mm / 24 h	> 10^6^	151	4201	N/A	5617	9390	4355	583	N/A	946,442,920
07.04.2010	21:00	Serra do Mar	Morro do Bumba (old landfill)	Niterói	Rio de Janeiro (RJ)	280 mm / 24 h	N/A	48	56	221	N/A	3200	60	2	N/A	146,587,277
01.01.2010	03:30	Serra do Mar	Enseada do Bananal	Ilha Grande (Angra dos Reis)	Rio de Janeiro (RJ)	440 mm / 36 h	55,600	31	2	N/A	N/A	N/A	7	1	N/A	180,668,819
11.01.2011	N/A	Serra do Mar	Cuiabá	Petrópolis	Rio de Janeiro (RJ)	273.8 mm / 24 h	> 10^6^	71	N/A	300	187	6956	N/A	N/A	N/A	1,932,584,390
			Vieira, Príncipe	Teresópolis		161.6 mm / 24 h		429	N/A		6727	9110				
			Rio Grande	Nova Friburgo		249 mm / 24 h		392	N/A		789	4528				
11.03.2011	05:00	Serra do Mar	Jacareí, Tingidor	Antonina, Morretes	Paraná (PR)	49 mm / h	N/A	4	21	N/A	2500	10000	211	12	N/A	76,095,510
22.02.2013	17:00	Serra do Mar	Ribeirão do Cágado, Rio Marcolino, Rio Pilões	Cubatão	São Paulo (SP)	118 mm / h	N/A	1	N/A	N/A	500	107	15	6	1	N/A
17.03.2013	20:30	Serra do Mar	Quitandinha	Petrópolis	Rio de Janeiro (RJ)	355 mm / 24 h	N/A	34	49	0	1074	1120	662	N/A	54	56,111,211
12.01.2014	23:00	Serra do Mar	Guarda mão, Palmital	Itaóca	São Paulo (SP)	150 mm / 6 h	N/A	23	4	0	20	332	110	10	3	5,578,975
29.03.2016	18:00	Serra da Mantiqueira	Córrego dos Araújos	Bom repouso	Minas Gerais (MG)	N/A	N/A	0	0	0	71	126	2	0	9	718,275
05.01.2017	N/A	Serra Geral	Mascarada river	Rolante	Rio Grande do Sul (RS)	270 mm / 3 h	N/A	0	0	0	0	1600	400	N/A	N/A	24,683,356
11.02.2017	20:00	Serra do Mar	Pedra Branca	Guaratuba	Paraná (PR)	128 mm / h	120,000	0	0	0	4	0	1	0	2	N/A
16.12.2020	N/A	Serra do Mar	Itajaí river basin	Presidente Getúlio, Ibirama	Santa Catarina (SC)	85 mm / 4 h	> 10^6^	13	35	6	175	1686	90	64	N/A	9,155,290

### Database compilation: challenges and constraints

The direct relationship of debris-flow events with high-intensity rainfall, which is also associated to flash floods and floods (Hungr et al. 2014), can undermine their recognition (Dowling and Santi 2014) and result in their misclassification and consequent underreporting (Guzzetti and Tonelli 2004), especially in the disaster databases. The misclassification is observed more often in high-magnitude events, such as the 2011 event in Rio de Janeiro, which is classified as a flood event in the S2ID and EM-DAT.

Another shortcoming found during cataloguing is related to the correct technical classification of debris flows in the databases, where most documented debris-flow events are, in fact, localized landslides. In S2ID, from 2012 to 2020, 61 debris-flow events are catalogued, although only three are indeed debris flows when analyzing their description and photos. In the IG database, from 1991 to 2018, 70 debris-flow events are recorded in the state of São Paulo, though only four can be considered debris flows. As pointed out by Dowling and Santi (2014), while technical literature usually correctly classifies the type of slope movement, such carefulness with terminology by journalistic reports, governmental and international aid documents (i.e., non-technical literature) is usually not observed.

Moreover, even though there can be several hydrogeomorphic processes associated to a debris flow, the phenomenon is responsible for greater direct damage and fatalities (Costa 1988; Coussot and Meunier 1996; Corominas et al. 2014). Economic losses involved with debris flows are, however, more uncertain, since the associated processes, especially flash floods and floods, are responsible for a larger radius of structural damage (Jakob et al. 2012; Álvala et al. 2019). In our debris-flow event dataset, due to the difficulties in separating the economic losses related to the main debris-flow event and the associated processes, the losses for the whole event are considered.

Furthermore, scientific publications and governmental reports tend to focus on larger events, which can potentially create a bias on the magnitude and frequency of debris-flow events in the country. This is also the case for the databases, which report only events with fatalities/economic losses, with those in remote areas or with smaller magnitudes often going unreported. Therefore, it must be highlighted that the compiled database is based on the available reported data, representing a baseline estimation of the damage that debris flows cause in Brazil.

### The impact of debris flows

Debris-flow events have caused at least 5.5 billion USD in direct economic losses during the considered period, and were responsible for over 5771 fatalities (including missing people) (Table 2). Debris-flow events have also caused the destruction of more than 11325 residences, 803 public and private buildings and 177 infrastructures. The combined number of homeless and displaced people is 211153, with 10104 people injured due to the phenomenon. According to our estimates, the average fatality rate per event is around 128 (total number of deaths/total number of debris-flow events) and the average economic loss per event is of ca. 122 million USD (total sum of economic losses/total number of debris-flow events).

**Table 2:** Analysis of debris-flow-related damage according to state. Fatalities include missing people. *Avg*, average; *N/A*, not available

State	Number of events	Economic losses (USD)	Fatalities	Avg. fatality/event	Avg. economic loss/event (USD)
Bahia (BA)	2	34,884,425	173	87	17,442,212
Ceará (CE)	1	N/A	91	91	N/A
Minas Gerais (MG)	3	718,275	286	95	359,138
Paraná (PR)	2	76,095,510	4	2	38,047,755
Rio de Janeiro (RJ)	13	3,517,256,731	4353	335	270,558,210
São Paulo (SP)	18	168,802,604	621	35	9,377,922
Santa Catarina (SC)	5	1,693,749,577	243	49	338,749,915
Rio Grande do Sul (RS)	1	24,683,356	0	0	24,683,356
**Total:**	45	5,516,190,478	5771	128	122,582,011

The largest event in terms of reported fatalities is the 1967 debris flow in Serra das Araras (Piraí, Rio de Janeiro state) (Fig. 1a), followed by the 2011 event in the mountain region of Rio de Janeiro (Tersópolis, Petrópolis) (Fig. 1b). These events have caused, respectively, 300 and 893 deaths and, at least, 1400 and 300 reported missing people. In terms of magnitude, the 1967 debris-flow event in Caraguatatuba (São Paulo state) has the largest reported magnitude (7,600,000 m^3^) (Fúlfaro et al. 1976).

The number of reported fatalities in the Caraguatatuba event is low (436) when compared to the magnitude and the destructive power of the event (Fig. 1c), and some studies suggest that the real figures are much higher (Listo et al. 2018). Both the Serra das Araras and Caraguatatuba events exhibit stark incompleteness of data despite their very extensive losses, which could be attributed to the lack of transparency from governmental data during the Brazilian military dictatorship (1964–1988), as some studies suggest (e.g., Ab’Sáber 1991; Sedrez and Maia 2014).

Considering that there are great uncertainties related to the 1967 events of Caraguatatuba and Serra das Araras, the 2011 event is the largest and most-destructive debris-flow event in Brazilian history, based on the more reliable reported figures. The 2011 catastrophe is also the largest when direct economic losses are considered, with reported losses estimated at ca. 1.9 billion USD in 2022 (Table 3).

**Table 3:** Analysis of debris-flow-related damage according to municipality. Fatalities include reported cases of dead and missing people. *N/A*, not available

Municipality/region	Number of events	Economic losses (USD)	Fatalities	Material losses (unit)
Além Paraiba (MG) and Volta Grande (MG) region	1	N/A	250	63
Antonina, Morretes (PR)	1	76,095,510	25	223
Bom repouso (MG)	1	718,275	0	11
Campos do Jordão (SP)	2	N/A	27	381
Caraguatatuba (SP)	1	63,449,964	436	400
Contagem (MG)	1	N/A	36	300
Cubatão (SP)	9	73,332,253	11	40
Guaratuba (PR)	1	N/A	0	1
Ilha Grande (RJ)	1	180,668,819	31	8
Itaóca (SP)	1	5,578,975	23	123
Lavrinhas (SP)	2	2,329,412	11	N/A
Maranguape (CE)	1	N/A	91	N/A
Niterói (RJ)	1	146,587,2770	269	62
Nova Friburgo (RJ)	1	1,932,584,390*	392**	N/A
Petrópolis (RJ)	6	322,416,245 + 1,932,584,390*	428**	1929
Rio de Janeiro (RJ)	4	1,010,547,344	804	> 1500
Rolante (RS)	1	24,683,356	0	400
Salvador (BA)	2	34,884,425	173	1401
Santos (SP)	2	24,112,000	102	67
Serra das Araras – Piraí (RJ)	1	N/A	1700	> 100
Teresópolis (RJ)	1	1,932,584,390*	429**	N/A
Timbé do Sul (SC)	1	71,311,286	16	N/A
Tubarão (SC)	1	666,840,081	40	N/A
Ubatuba (SP)	1	N/A	11	30
Vale do Itajaí region (SC)	3	946,442,920	187	5158

*Economic losses associated to the 2011 debris flow event, undistinguished between cities

**+ 300 missing people, undistinguished between cities

### Geographic distribution

**Fig. 2:**
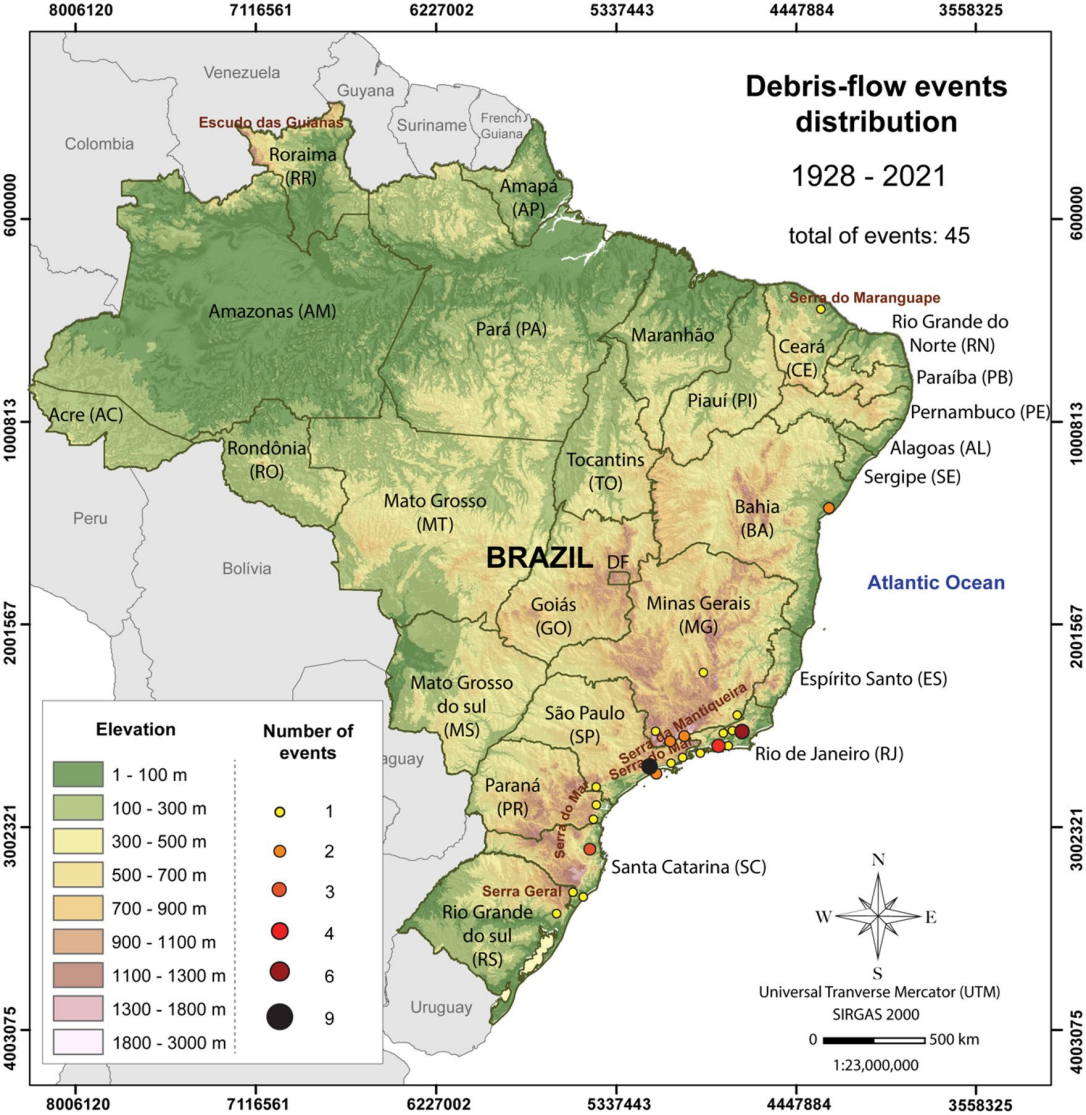
Geographic distribution of reported debris-flow events in Brazil. The reported events are more often associated to the mountain ranges of Serra do Mar, Serra da Mantiqueira, and Serra Geral. The underlying Digital Elevation Model (DEM) is created using GTOPO30 data retrieved from Earth Explorer (USGS 2021)

Figure 2 shows the spatial distribution of debris-flow events in the Brazilian territory. In total, 64.5% of the recorded debris-flow events occurred in the Serra do Mar Mountain range, followed by Serra da Mantiqueira (13.3%) and Serra Geral (13.3%). The southeast region of Brazil is the most affected by debris flows, both in terms of number of events and socioeconomic losses (Table 4). This can be related to the highest population density in the southeast (especially in the states of Rio de Janeiro and São Paulo), the intense urbanization of the coast and the presence of two mountain regions: Serra do Mar and Serra da Mantiqueira.

**Table 4:** Analysis of reported debris-flow-related damage according to region. Fatalities include missing people. For state abbreviations, please refer to Fig. 2. *Avg.*, average

Regions	Number of events	Economic losses (USD)	Fatalities	Avg. fatality per event	Avg. economic loss per event (USD)
North (AC, AM, AP, PA, RO, RR, TO)	0	0	0	0	0
Northeast (AL, BA, CE, MA, PB, PE, PI, RN, SE)	3	34,884,425	264	88	11,628,142
Center-West (DF, GO, MS, MT)	0	0	0	0	0
Southeast (ES, MG, RJ, SP)	34	3,686,777,610	5260	155	102,410,489
South (PR, RS, SC)	8	1,794,528,444	249	31	224,316,056

São Paulo is the state with the highest number of reported debris-flow events, followed by Rio de Janeiro and Santa Catarina (Table 2). The state of Rio de Janeiro is, by far, the most impacted by debris flows, with 4353 fatalities in the last 100 years and ca. 3.5 billion USD in economic losses, which corresponds to approximately 75% of all fatalities and 64% of the economic losses reported in the whole country. Rio de Janeiro also shows the highest fatality rate per event than any other state, averaging at 335 deaths per event, while Santa Catarina shows the highest economic losses per event, averaging at ca. 339 million USD per event (Table 2). These numbers are based on reported numbers and the available data.

Among the cities most affected by debris flows, Cubatão (São Paulo state) and Petrópolis (Rio de Janeiro state) stand out with the highest numbers of events in the last 100 years (9 and 6 events, respectively), whereas the city of Rio de Janeiro, Petrópolis, and Tubarão (Santa Catarina) show the highest economic losses (Table 3). Serra das Araras (Piraí), the city of Rio de Janeiro, Petrópolis, and Caraguatatuba show the largest number of fatalities (Table 3).

### Temporal and seasonal analysis

**Fig. 3:**
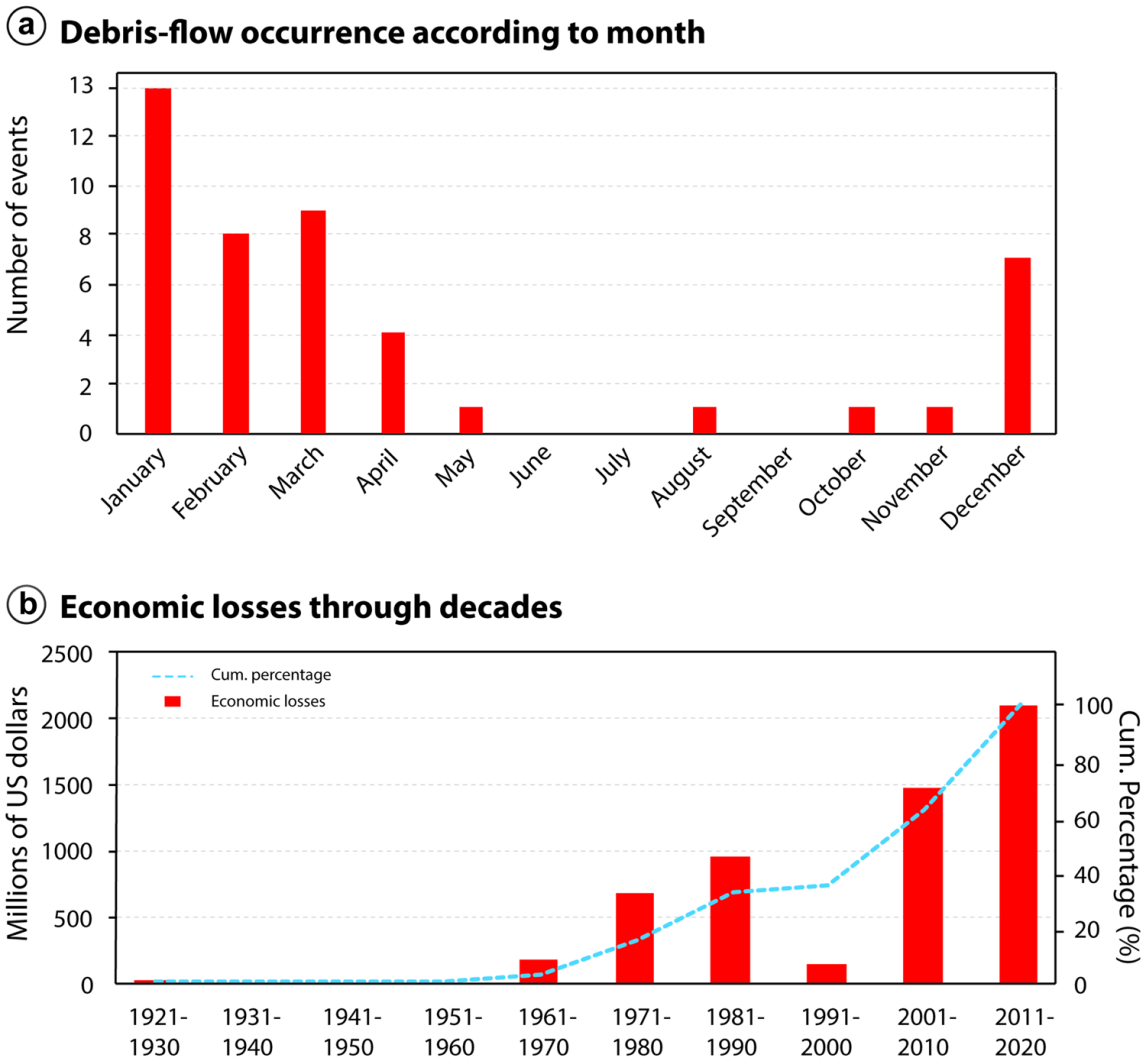
Seasonal and temporal occurrence of debris-flow events and associated economic losses. **a** Monthly distribution of debris-flow events in Brazil. Debris flows are more common in summer months (December–March), which show highest rainfall indices. **b** Temporal distribution of economic losses related to debris-flow events. There is an increase in debris-flow-related economic losses in the last two decades (2000–2020), which could be related to a better reporting and monitoring, as well as to the increase in urbanization in the country

Debris-flow events are more common during summer season (December–March), which is the wettest season in Southeast Brazil, where Serra do Mar and Serra da Mantiqueira are located. Figure 3a shows the seasonal distribution of events, with January having the largest number of reported debris-flow events, followed by March, February and December. The phenomenon can occasionally occur during winter in the Southeast, due to abnormally rainfall events (Seluchi et al. 2011) as the one in Campos do Jordão (São Paulo state) in August 1972 at Serra da Mantiqueira (Table 3).

**Fig. 4:**
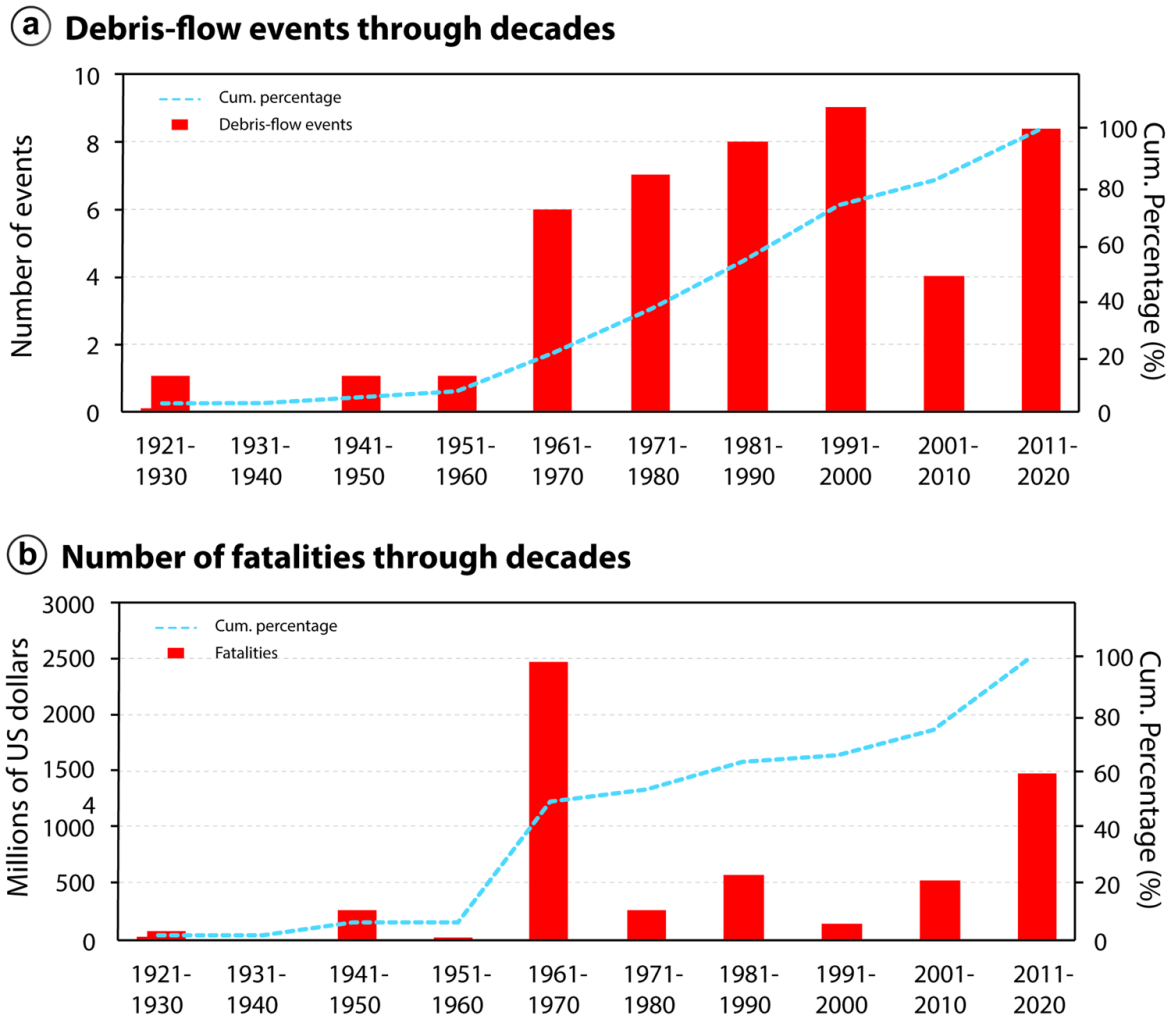
Temporal analysis of debris-flow events and human-related damage. **a** Number of debris-flow events, which have been steady since the 1960s, averaging at about seven events per decade. **b** Casualties related to debris flows in Brazil. The years of 1967 and 2011 stand out, due to the high-magnitude events of Serra das Araras and Caraguatatuba (1967), and Teresópolis, Petrópolis and Nova Friburgo (2011)

The economic losses associated to debris-flow events have been increasing since 1920, although no reports are available for the decades of 1930s, 1940s, and 1950s (Fig. 3b). In the 1990s, a sharp decrease in reported economic losses is observed, which is also followed by a decrease in the number of fatalities (Fig. 4b), although not by the number of events (Fig. 4a). The general trend, however, is a growth in the associated economic losses over the two more recent decades, which can be related to a better reporting of disasters, as well as to the increase in urbanization in the country.

As for the number of reported events, there has not been a significant increase in recent decades, with the frequency averaging at about seven per decade since the 1960s. Prior to 1960s, the documentation of debris-flow events is scarcer, which can be associated to lower levels of urbanization and, consequently, a lower societal impact. The number of fatalities along the years has also been steady since the 1960s, with the decades of 1960s and 2010s standing out as those with the highest number of fatalities, particularly in the years of 1967 and 2011 (Fig. 4b). These 2 years are characterized by the high-magnitude debris-flow events in Caraguatatuba and Serra das Araras (1967), and Teresópolis, Petrópolis and Nova Friburgo (2011).

### Rainfall analysis

**Fig. 5:**
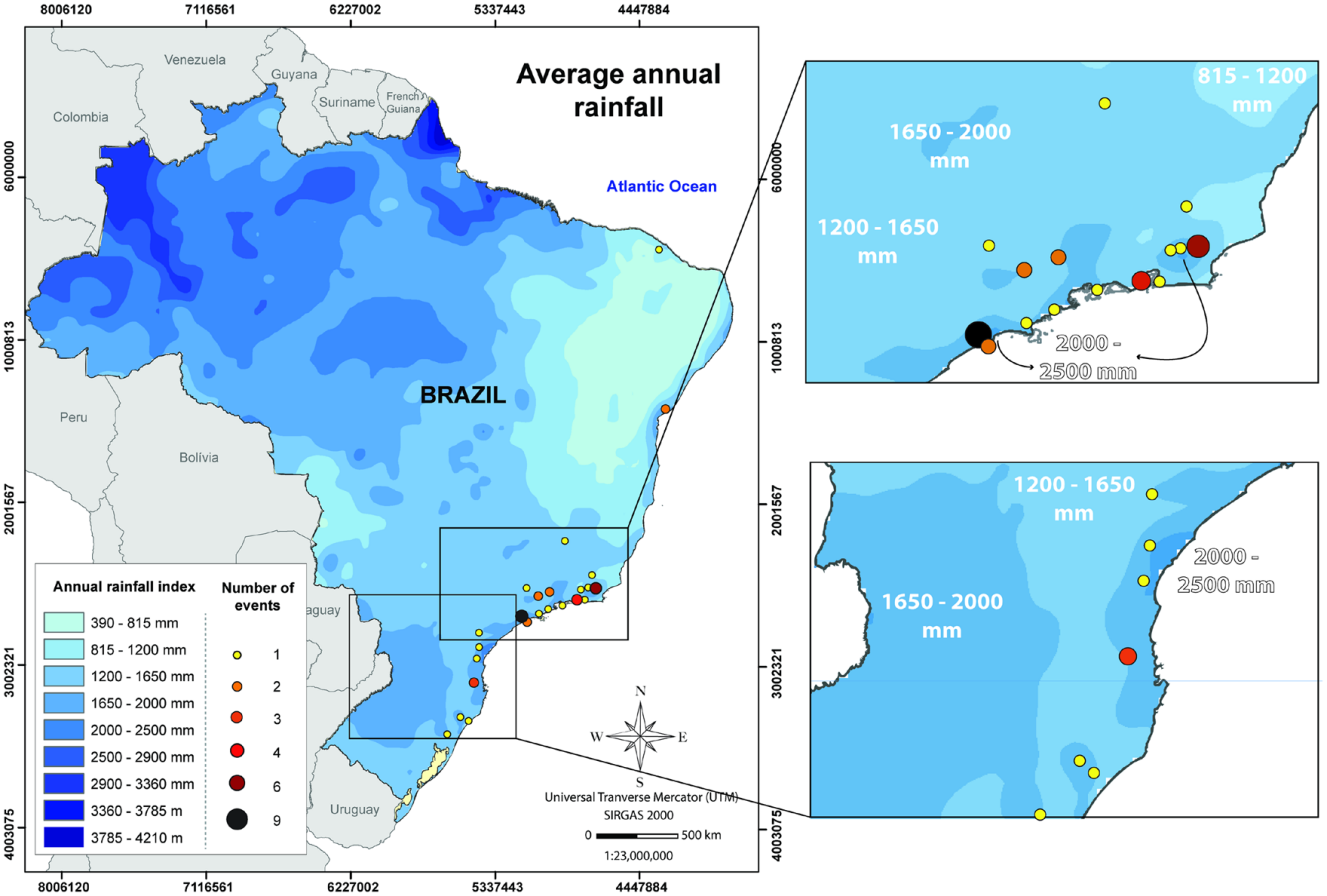
Average rainfall indices for Brazil, based on climate data from Brazil’s pluviometric atlas (CPRM 2021). Debris flows generally occur in areas with average annual rainfall higher than 1650 mm

Figure 5 shows the average annual rainfall indices for Brazil plotted against reported debris-flow events. Debris flows are concentrated in regions with high rainfall rates (> 1600 mm annually) that are also associated with mountain regions, highlighting the strong association between hilly areas and precipitation for their occurrence. In areas with very-high annual precipitation (> 2500 mm) and no debris-flow records, such as the Northern region of Brazil, the relatively flatter terrain or the remoteness of the mountain areas (e.g., Escudo das Guianas, at the border with Venezuela – Fig. 2) can be associated to the lack of recorded events.

In our catalogue, high-resolution rainfall information was often deficient and, for the most cases, only daily rainfall was available (Table 1). Comparing the 24-h accumulated rainfall with reported data of magnitude, economic losses, and fatalities, we can observe a very weak relationship between rainfall and these variables. The relationship between 24-h accumulated rainfall and magnitude, fatality number, and economic losses show, respectively, a Spearman correlation coefficient of −0.01 (*p*-value of 0.52), 0.01 (*p*-value of 0.51), and −0.25 (*p*-value of 0.81).

When peak rainfall intensity is considered, a slightly stronger positive correlation between magnitude and number of fatalities is observed. The relationship between hourly rainfall and magnitude shows a positive Spearman correlation coefficient of 0.7 (*p*-value of 0.91), while the relationship between fatality number and hourly rainfall shows a positive Spearman correlation coefficient of 0.36 (*p*-value of 0.81). The correlation between hourly rainfall and economic losses, on the other hand, is weaker, with a negative Spearman correlation coefficient of −0.2 (*p*-value 0.63) (see online resources for supplementary information about the correlation of rainfall data and debris-flow magnitude, fatality toll and economic losses).

The available rainfall data, therefore, is not sufficient to indicate a clear relationship between rainfall indices and economic losses, fatality number and magnitude. These results can indicate that the damage related to debris-flow events is not only a function of rainfall, but also to social (e.g., urbanization levels, occupation of risk areas) and geomorphic (e.g., vegetation cover, catchment and channel slope, on-channel material) factors.

Even though hourly rainfall (peak intensity) showed a stronger correlation with magnitude and the number of fatalities, the small sample space of events with complete data of all the considered variables challenges a concrete conclusion about their relationship. It is expected, however, that the more intense the rainfall, the larger the event and, consequently, the larger the associated damage.

### Evaluation of the debris-flow impacts

**Fig. 6:**
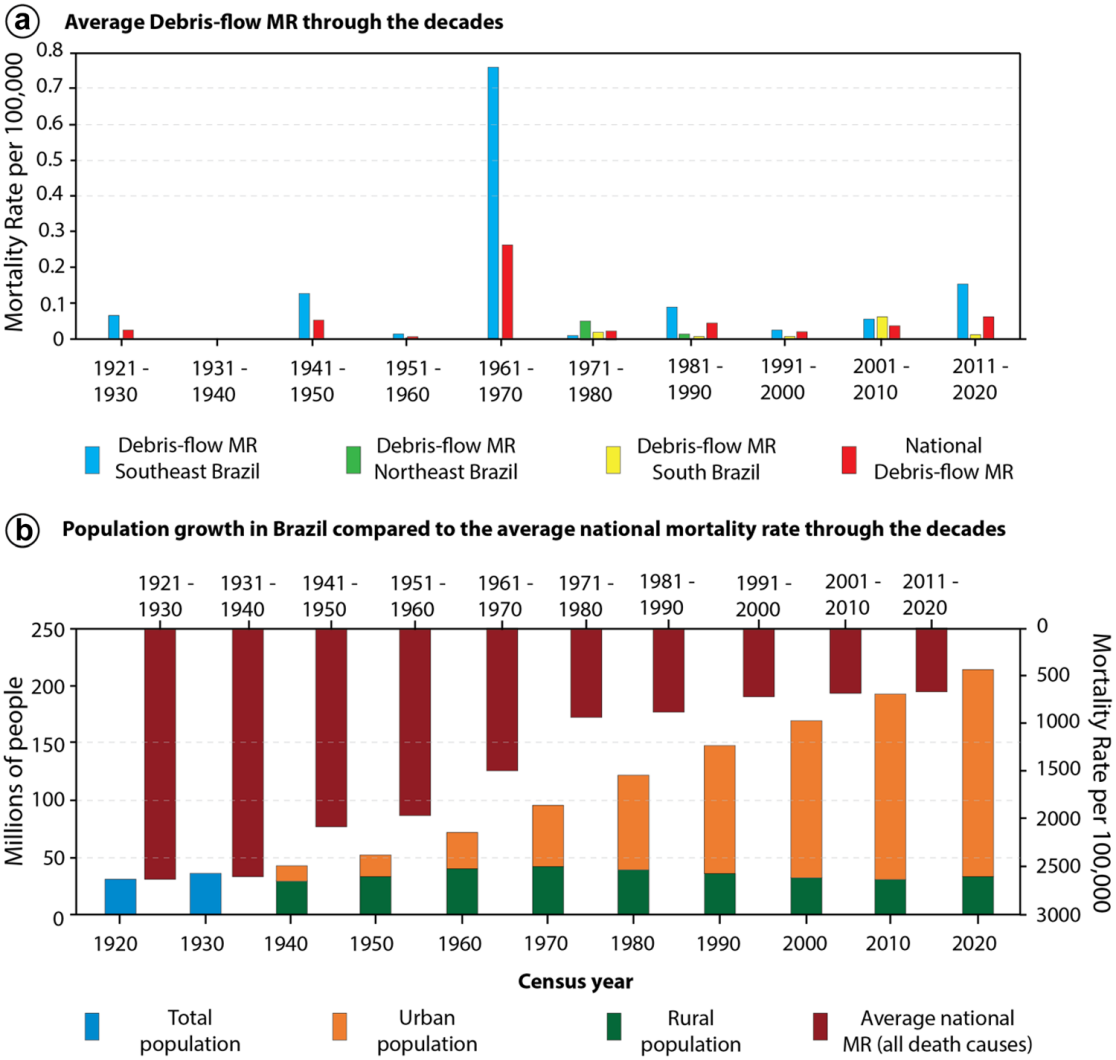
Mortality rate (MR) and demographic analysis. **a** Average national debris-flow MR (red bar) through the decades, compared to the debris-flow MR of the Southeastern (blue), Northeastern (green), and Southern (yellow) region. **b** Populational growth of Brazil according to census year, compared to the average mortality rate through the decades, comprising all death causes (dark red bar). The number of people living in urban areas has been steadily increasing since 1960. The demographic and average MR data for Brazil is retrieved from IBGE (2019, 2021)

Figure 6a shows the average MR of debris flows per 100,000 habitants in Brazil for every decade since 1920 to 2020, whereas the average national MR (combining all death causes) is shown in Fig. 6b, plotted against the population growth of Brazil. The results indicate that while the average national MR has been going down since 1920, the national MR of debris flows has been fairly steady through the decades. The comparison between the declining average national MR with the steady debris-flow MR can suggest that while there have been several advances in public health and public security policies in Brazil since 1920, the same is not observed for debris-flow prevention and mitigation measures.

Southeast Brazil (SEB) exhibits the highest debris-flow MR, with debris-flow-related fatalities reported in all decades since 1920, except for the 1930s (Fig. 6a). The average MR in SEB ranged from 0.06 in 1920s to 0.76 in the 1960s, decreasing in the 1970s (0.004) and increasing again in the last decade (0.15). In South Brazil (SB), debris-flow-related fatalities are recorded every decade since the 1970s, with the highest average debris-flow MR in the 2000s (0.06), due to the debris-flow event in the Itajaí river basin in 2008 (Table 3). In Northeast Brazil (NEB), only in the 1970s and 1980s the region reported debris-flow-related deaths, with the highest debris-flow MR in the 1970s (0.05).

**Fig. 7:**
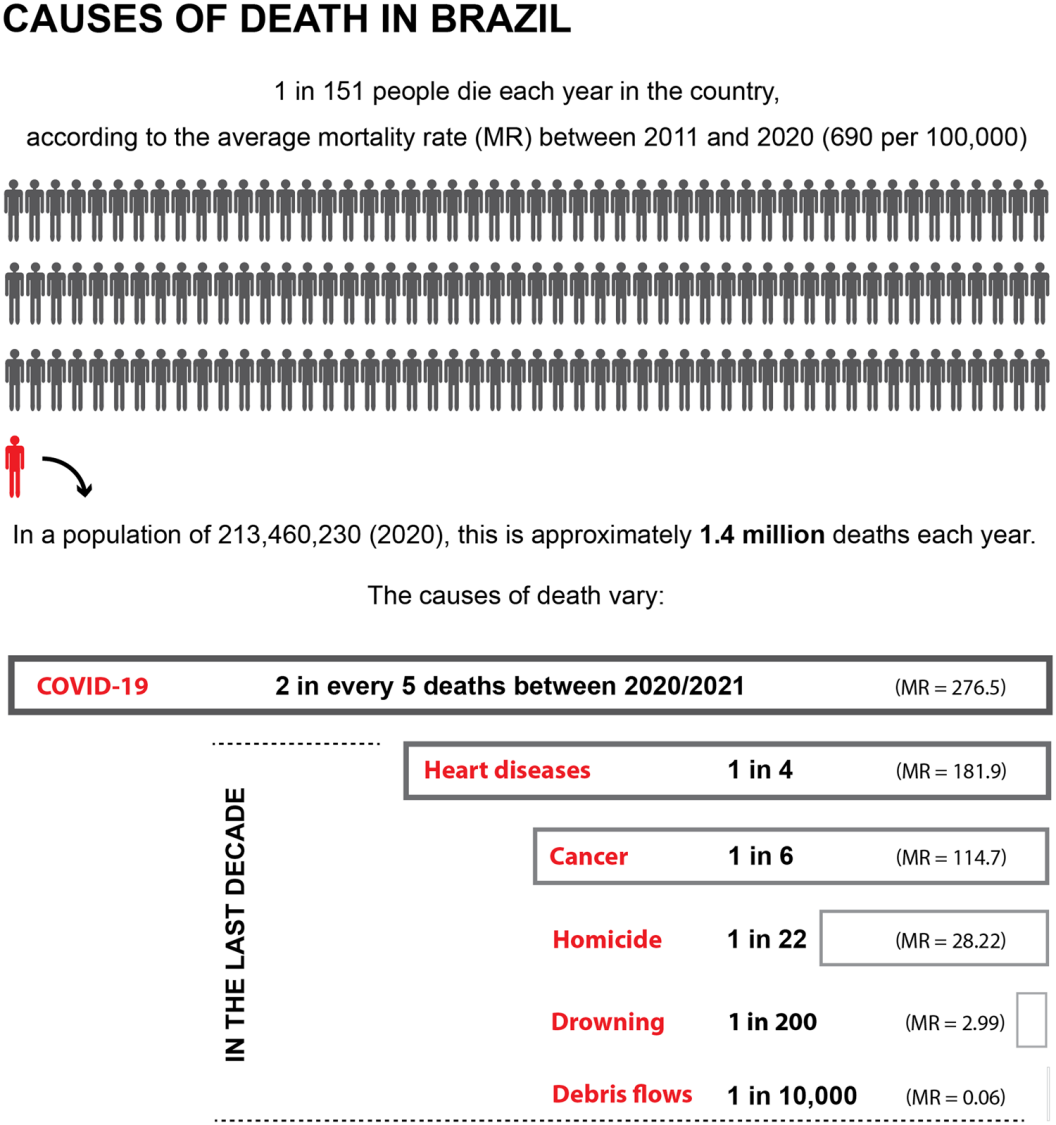
Mortality rate (MR) per 100,000 people per year of different death causes in Brazil. The average mortality rates for the last decade (2011–2020) are based on data from the Global Burden of Diseases (Vos et al. 2020). The MR of coronavirus in Brazil is based on data from the Brazilian Health Ministry (Brasil 2021c). The figure is based on Strouth and McDougall (2021)

The debris-flow MR in the country compared to the MR of other diseases and human-induced causes is shown in Fig. 7, considering the average MR values for the last decade (2011–2020) according to the Global Burden of Diseases (Vos et al. 2020). Fatalities related to debris flows in Brazil are rather rare when compared to other death causes, with approximately 127 deaths per year during the last decade, while homicides and drowning cause each year approximately 61000 and 6380 deaths per year, respectively (Fig. 7). The primary cause of death in Brazil is diseases, followed by public violence, with COVID-19-related deaths representing the leading cause of deaths between 2020 and 2021.

**Fig. 8:**
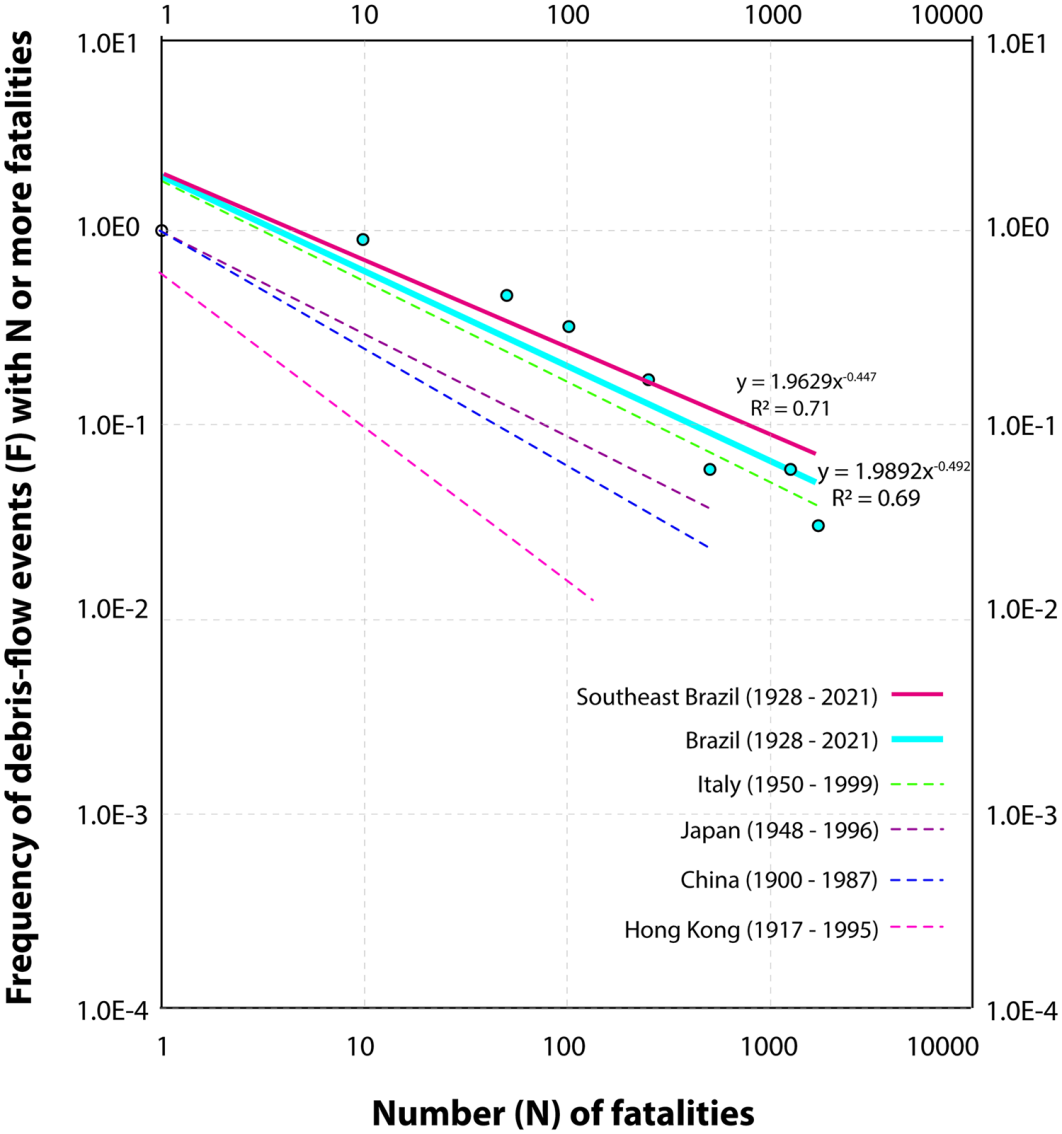
F-N curves of debris-flow events in Brazil compared to other countries. Debris flows show a higher societal impact in Brazil than landslide events (also comprising debris flows) in China (Tianchi 1989), Japan (Cascini et al. 2008), Hong Kong (Ho and Ko 2009) and Italy (Guzzetti 2000)

Furthermore, according to the F-N curves for debris flows in the country (Fig. 8), an event with the fatality number of 1200 or more, such as the one in 2011 in Rio de Janeiro, has a probability of occurring every 50 years, if no mitigative measures are adopted, and a debris flow with 10 or more fatalities has a probability of occurring every 3 years.

**Fig. 9:**
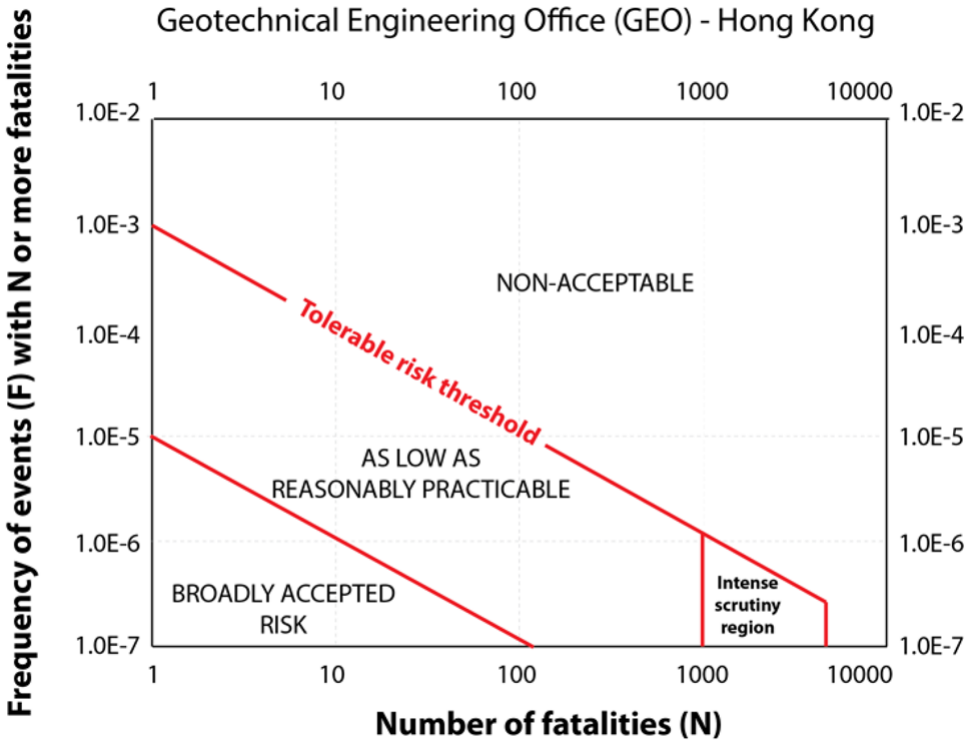
Societal risk thresholds adopted by Hong Kong, which commissioned the creation of the criterions specifically for landslides (ERM 1998)

When the consequences of debris-flow events in Brazil is compared to the consequences of landslide events in other countries, which also include debris flows, the phenomenon in Brazil stands out showing a higher societal impact than in China (Tianchi 1989), Japan (Cascini et al. 2008) and Hong Kong (Ho and Ko 2009), and a similar impact to that of Italy (Guzzetti 2000) (Fig. 9). As already observed using the debris-flow MR to analyze the consequences in Brazil, SEB exhibits a higher probability of fatal debris-flow events than the national average (Fig. 9), showing a gentler slope (−0.447) when compared to the whole nation (−0.492), consolidating its status as the most susceptible region to the phenomenon.

In our analysis, however, we focus only on debris-flow events, which tend to cause a higher number of fatalities and are less frequent than localized fatal landslides (Corominas et al. 2014), which could potentially impact the slope of the F-N curve. Moreover, Japan, Italy, and Hong Kong are much smaller in territorial area than Brazil and SEB, which can also potentially impact the probability of fatal events due to the scale effect.

## Discussions

F-N plots are commonly applied in landslide studies worldwide (e.g., Macciotta et al. 2016; Keller 2017; Zhang et al. 2019; Strouth and McDougall 2021; among others), even though they are not universally acknowledged as a good indicator of risk (Evans and Verlander 1997; Strouth and McDougall 2021). F-N plots have also been applied in the establishment of thresholds of what is deemed as an acceptable risk by society for different types of natural hazards, including specifically for landslides (Malone 2005; Strouth and McDougall 2021).

Hong Kong, through the country’s Geotechnical Engineering Office (GEO), established landslide risk thresholds (ERM 1998) (Fig. 9), which have been adopted by some countries (e.g., Australia, AGS 2007; Western Canada, Porter and Morgenstern 2013), though, as pointed out by Strouth and McDougall (2021), to no great success taking Canada as an example.

F-N curves can potentially be applied in risk analysis studies in cities with extensive historical incidence of debris-flow events in Brazil, such as Petrópolis and Cubatão, providing a good estimation of the probability of fatal events occurrence, which can later subsidize decisions about preventive measures and potentially be incorporated in municipal laws to guide urban occupation in areas prone to the phenomenon. While F-N curves can be useful, the adoption of risk thresholds, such as the one from GEO (ERM 1998), can be more challenging. Despite similar climate and hydrogeomorphic process dynamics (Ho and Ko 2009; Lacerda 2007), especially at SEB (sub-tropical climate), economic and cultural differences on the perception of landslides and debris-flow risk are some of the factors that are not easily transferable (Strouth and McDougall 2021).

For instance, between 2016 and 2017, the Hong Kong Government budget for disaster prevention and preparedness was approximately 396 million USD, with 165 million USD for landslide preventive measures (Sim et al. 2018). CEMADEN in Brazil had an annual budget upon its creation of 14 million USD in 2012–2013, which has been successively slashed through the years, reaching approximately 3.7 million USD in 2019–2020 (Brasil 2021b). In addition to very different budgets implemented for natural disasters prevention, there are scale effects in the creation of risk thresholds over F-N plots that should be considered, as the *x*-axis (N) is affected by the size of the population affected, and the *y*-axis (F) by the return period of the phenomenon (Strouth and McDougall 2021). Comparing the probability of fatal debris-flow events in Brazil with other countries using F-N plots is also subjected to the scale effect, especially when we compare it to Italy and Hong Kong.

Nonetheless, the use of the F-N plot provides a good indication of the risk that the phenomenon represents in Brazil, especially at SEB, and the steady debris-flow MR through the decades further shows that little has been made to reduce and prevent their negative impacts in the last 100 years. The temporal and spatial distribution of debris-flow events is a primordial step to understanding the impact of the phenomenon and provides useful information for the definition of areas where mitigative measures must be implemented. Considering that Brazil’s population growth is most concentrated at coastal cities in southeast (Londe et al. 2018), which are also the “hotspots” for debris-flow events, disaster prevention measures can and should be implemented, such as local-scale risk analysis, early warning systems and installation of retention structures.

The outlook, however, is not promising. The results of the consistent underfunding of CEMADEN and the discontinuation of important disaster prevention programs have been recently seen in the flood and landslide event that struck Petrópolis in February 2022, where no warning was issued, causing hundreds of fatalities, despite extensive hazard and risk mapping of the municipality. As our study shows, the city is one of the most historically affected by debris-flow events and is characterized by high rainfall indices and by the occupation of hilly areas by residences and industries.

While rainfall dynamics in the country can vary greatly according to region (Seluchi et al. 2011; Marengo et al. 2021), generally speaking a combination of antecedent and high-intensity peak precipitation is the main rainfall pattern that triggers debris-flow events (Kobiyama et al. 2010; Debortoli et al. 2017). However, as pointed out by Borga et al. (2014), the past is not necessarily the key to the future, especially when land use changes and global warming  can potentially alter precipitation patterns, affecting the dynamics of hydrogeomorphic processes in a region or country (Westra et al. 2014; DeBortoli et al. 2017; Marengo et al. 2021).

Furthermore, studies in regions that can also be susceptible to debris-flow initiation, but no fatal events have been recorded so far, are valuable, both considering the population increase trend and the comprehension of debris-flow dynamics in different geological-geomorphological settings in Brazil. Particularly, more studies considering the hydrogeomorphic aspects of catchments in regions such as Serra do Araripe in Pernambuco state (Peulvast et al. 2011), Escudo das Guianas in Amapá state and in the hilly areas of the central region of Brazil (Mato Grosso and Mato Grosso do Sul states) are welcomed. The lack of recorded debris-flow events in these areas can be linked to less frequent heavy-rain events and to lower levels of occupation of hilly areas when compared to Southeast and South Brazil.

## Conclusions

Cataloguing and estimating the consequences that a natural hazard represents based on past events is one of the most effective methods to provide reasonable damage assessments to the society, even though incompleteness of data and lack of minor events are common challenges that have to be minded. Our historical analysis shows that debris-flow events are concentrated mostly in the Serra do Mar and Serra da Mantiqueira mountains in the Southeast region of Brazil, where population density is higher and occupation of hilly areas is more common. Between 1920 and 2021, 45 debris-flow events were responsible for over 5771 fatalities and 5.5 billion USD in economic losses. Petrópolis (Rio de Janeiro State), the city of Rio de Janeiro, Cubatão (São Paulo State), and the Vale do Itajaí region (Santa Catarina State) are the most affected areas by debris flows in Brazil, with an extensive history of fatal and destructive events. These regions are characterized by high rainfall indices, especially during summer, and urban and industrial areas near or at mountain areas.

The application of F-N plots shows that the phenomenon represents great risk to the Brazilian society, with a probability of a debris-flow event with a fatality rate of over 1200 people occurring every 50 years. Based on the average debris-flow MR for the last decade (2011–2020), 1 in every 10,000 deaths was due to the phenomenon, which is low when compared to other deaths causes, such as drowning (1 in 200) and homicide (1 in 22). However, contrasting with the evolution of the national MR (all death causes), which has been decreasing in the last 100 years, the steady MR for debris-flow through the decades indicates that little has been made to reduce the negative impacts of the phenomenon in the considered period.

Finally, as landslides are the main triggering mechanism of debris flows in Brazil, the creation of a national landslide inventory can help to identify patterns that lead to the phenomenon in catchments, further aiding the characterization of debris-flow dynamics. The use and widespread availability of GIS technology can facilitate the creation of a collaborative database, with researchers and technicians from different institutions responsible for the update as new data is available and mapped. Efforts in this regard have been recently made by Brazilian research groups and researchers (e.g., Uehara et al. 2020; Dias et al. 2021; Osako 2021; among others).

## Supplementary Information

Below is the link to the electronic supplementary material.

Supplementary file1 (DOCX 16 KB)

Supplementary file2 (DOCX 16 KB)

Supplementary file3 (XLSX 38.6 KB)

Supplementary file4 (XLSX 3004 KB)

## Footnotes

1 According to the definition of the United Nations Finance Center for South-South Cooperation (UN FCSSC, 2022).
