# Supplementary material for: The consequences of debris flows in Brazil: a historical analysis based on recorded events in the last 100 years
Source: Landslides. 2022 Dec 13;20(3):511–29. doi: 10.1007/s10346-022-01984-7 (PMC9745771; doi:10.1007/s10346-022-01984-7)
Supplement: Supplementary file 1 — Supplementary file1 (DOCX 16 KB) [file 10346_2022_1984_MOESM1_ESM.docx]

Manuscript title: **The consequences of debris flows in Brazil: a historical analysis based on recorded events in the last 100 years**

^1,2^Victor Cabral, ^1^Fábio Reis, ^1^Vinicius Veloso, ^1^Claudia Correa, ^1,2^Caiubi Kuhn, ^2^Christiane Zarfl

^1^ Applied Geology Department, Earth and Exact Sciences Institute, São Paulo State University – UNESP. Address: Av. 24A, 1555 – Rio Claro, São Paulo, Brazil.

^2^ Geo- und Umweltforschungszentrum (GUZ), University of Tübingen. Address: Schnarrenbergstraße 94 – 96, Tübingen, Germany

**Supplementary Information – Definition of disaster type according to different disaster database**

Hydrogeomorphic processes classification by the Center for Research on the Epidemiology and Disasters (CRED) and the Brazilian Code of Disasters (COBRADE)

| **CRED** | | **COBRADE - Based on Varnes (1978)** | |
| --- | --- | --- | --- |
| *Process* | *Definition* | *Process* | *Definition* |
| Avalanche | A large mass of loosened earth material, snow, or ice that slides, flows or falls rapidly down a mountainside under the force of gravity.  ·Snow Avalanche: Rapid downslope movement of a mix of snow and ice;  ·Debris Avalanche: The sudden and very rapid downslope movement of unsorted mass of rock and soil. There are two general types of debris avalanches - a cold debris avalanche usually results from an unstable slope suddenly collapsing whereas a hot debris avalanche results from volcanic activity leading to slope instability and collapse. | Landslide | Process that can occur in natural and man-made slopes, triggered by water saturation generated by meteoric water infiltration or due to anthropogenic activities. Landslides are movements of soil and/or rock, with relatively short duration, with a well-defined rupture surface, which gravity center moves down and out of the slope. Frequently, the first signs of these movements are fissures in the slope. |
| Landslide | Any kind of moderate to rapid soil movement, including lahar, mudslide, debris flow. A landslide is the movement of soil or rock controlled by gravity and the speed of the movement usually ranges between slow and rapid, but not very slow. It can be superficial or deep, but the materials have to make up a mass that is a portion of the slope or the slope itself. The movement has to be downward and outward with a free face. | Debris flow | Rapid flow of a mass of soil and/or rock and/or man-made material, with the evolution in a river channel resembling a viscous liquid, with internal deformation and shear zones. The material that comprises a debris flow is usually mobilized in great volumes, with high destructive power and extensive damage radius. |
| Flash flood | Rapid inland floods due to intense rainfall A flash flood describes sudden flooding with short duration. In sloped terrain the water flows rapidly with a high destruction potential. In flat terrain the rainwater cannot infiltrate into the ground or run off (due to small slope) as quickly as it falls. Flash floods typically are associated with thunderstorms. A flash flood can occur virtually at any place. | Flash flood | The phenomena can be defined as a high energy and concentrated superficial flow, associated or not to fluvial processes. Flash floods can occur along roads constructed upon old fluvial courses with high hydraulic gradient or in steep areas. |
