# Supplementary material for: The consequences of debris flows in Brazil: a historical analysis based on recorded events in the last 100 years
Source: Landslides. 2022 Dec 13;20(3):511–29. doi: 10.1007/s10346-022-01984-7 (PMC9745771; doi:10.1007/s10346-022-01984-7)
Supplement: Supplementary file 2 — Supplementary file2 (DOCX 16 KB) [file 10346_2022_1984_MOESM2_ESM.docx]

Manuscript title: **The consequences of debris flows in Brazil: a historical analysis based on recorded events in the last 100 years**

^1,2^Victor Cabral, ^1^Fábio Reis, ^1^Vinicius Veloso, ^1^Claudia Correa, ^1,2^Caiubi Kuhn, ^2^Christiane Zarfl

^1^ Applied Geology Department, Earth and Exact Sciences Institute, São Paulo State University – UNESP. Address: Av. 24A, 1555 – Rio Claro, São Paulo, Brazil.

^2^ Geo- und Umweltforschungszentrum (GUZ), University of Tübingen. Address: Schnarrenbergstraße 94 – 96, Tübingen, Germany

**Supplementary Information – Bibliographic search results**

Bibliographic search results. Note: The number of publications for each keyword does not necessarily reflect the number of events that have occurred in Brazil since the beginning of the 20^th^ century.

| **Bibliographic search tool** | **Keyword** | **Number of Publications** |
| --- | --- | --- |
| SCOPUS (Elsevier) | Debris flows | 99 |
|  | Mudslides | 4 |
|  | Flash floods | 59 |
|  | Floods | 1,950 |
|  | Landslides | 425 |
| Web of Science (Clarivate Analytics) | Debris flows | 94 |
|  | Mudslides | 12 |
|  | Flash floods | 50 |
|  | Floods | 787 |
|  | Landslides | 451 |
| Periódicos CAPES | Debris flows (*corrida/fluxo de detritos*) | 131 |
|  | Mudslides (*corrida/fluxo de lama*) | 3 |
|  | Flash floods (*enxurrada*) | 1,607 |
|  | Floods (*inundação*) | 1,750 |
|  | Landslides (*escorregamentos*) | 216 |
